# Calibration of spectra in presence of non-stationary background using unsupervised physics-informed deep learning

**DOI:** 10.1038/s41598-023-29371-9

**Published:** 2023-02-07

**Authors:** Alessandro Puleio, Riccardo Rossi, Pasqualino Gaudio

**Affiliations:** Department of Industrial Engineering, University of Rome “Tor Vergata”, Via del Politecnico 1, 00133 Rome, Italy

**Keywords:** Applied physics, Optical physics, Techniques and instrumentation

## Abstract

Calibration is a key part of the development of a diagnostic. Standard approaches require the setting up of dedicated experiments under controlled conditions in order to find the calibration function that allows one to evaluate the desired information from the raw measurements. Sometimes, such controlled experiments are not possible to perform, and alternative approaches are required. Most of them aim at extracting information by looking at the theoretical expectations, requiring a lot of dedicated work and usually involving that the outputs are extremely dependent on some external factors, such as the scientist experience. This work presents a possible methodology to calibrate data or, more generally, to extract the information from the raw measurements by using a new unsupervised physics-informed deep learning methodology. The algorithm allows to automatically process the data and evaluate the searched information without the need for a supervised training by looking at the theoretical expectations. The method is examined in synthetic cases with increasing difficulties to test its potentialities, and it has been found that such an approach can also be used in very complex behaviours, where human-drive results may have huge uncertainties. Moreover, also an experimental test has been performed to validate its capabilities, but also highlight the limits of this method, which, of course, requires particular attention and a good knowledge of the analysed phenomena. The results are extremely interesting, and this methodology is believed to be applied to several cases where classic calibration and supervised approaches are not accessible.

## Introduction

Spectroscopy is among the most relevant techniques to extract information from the interaction of electromagnetic waves with matter^1–3^. Absorption spectroscopy, laser-induced fluorescence, Raman spectroscopy, and laser-induced breakdown spectroscopy are only a few of the vast number of techniques that, by analysing a spectrum, allow one to probe the medium, providing information about its atoms, molecules, crystals, etc^4–7^. Their properties have made spectroscopy a leading technology not only in physical science but also in industries, medicine, art, etc. For example, Raman spectroscopy finds applications in everything: from medicine^8,9^, by allowing even cancer diagnosis^10^, to art and cultural heritage^11^, from food science to environmental and explosive monitoring^12–15^. Laser-Induced Breakdown Spectroscopy (LIBS) is also widely used to analyse the atomic composition of matter and recently, also due to the development of machine learning algorithms, it has been applied in several fields of science and industries^16–18^, such as food^19^, chemical^20^, pharmaceutical^20^, and medicine^20^. The same applies for absorption spectroscopy, that changing the electromagnetic radiation wavelength goes from radio wave to X-ray, leading to a huge number of techniques (nuclear magnetic resonance spectroscopy, IR absorption spectroscopy, UV–vis absorption spectroscopy, etc.) and applications^21–23^. Being most of them no-contact measurements, in extreme environments, the use of light and its interaction with matter is the only way to make diagnostics. An example is the plasma in nuclear fusion reactors that reaching temperature of the order of 150 million degrees it can be analysed only with external measurements (for example, magnetic coils), while the core of the plasma is analysed by studying the interaction of electromagnetic waves with the plasma^24,25^. It involves that in a nuclear fusion reactor there is a huge number of spectroscopic diagnostics^26^, used, for example, to monitor plasma ions concentration, the presence of impurities, observe some edge instabilities, etc^25,27,28^. A similar argument applies, of course, to space and atmosphere science^29–31^.

Optical spectroscopy techniques rely on studies of the resulting spectra from light-matter interactions. In addition to the accurate work that scientists need to do for hardware selection, alignment, control of the environmental conditions to ensure specific properties of the matter, etc. There is also a huge work required to develop efficient algorithms able extract the desired pieces of information from a measured spectrum^32,33^. Some spectroscopy problems can be conceptualised as follows. Suppose to have a spectroscopic technique that allows one to measure the spectrum *I*_*d*_ and we want to evaluate a quantity *y* that is linked with the spectrum property such that *y* = *f(I*_*d*_*)*. In ideal cases, *f(I*_*d*_*)* is usually known (imagine, for example, the concentration measurement of a gas by using absorption spectroscopy, where you know the absorption cross-section spectrum of chemical^34^). Unfortunately, in real cases *f(I*_*d*_*)* is not known a priori and is difficult to estimate, even with numerical simulations, for several reasons. At first, components are not ideal (for example, the optics have properties that must be calibrated). Second, even small misalignment can change the optics response. Moreover, spectra are usually the results of not only the spectrum *I*_*d*_ relative to the physical effect that we are considering, but it contains spurious effects due to other minor (hopefully) phenomena, such as background light, secondary emission or absorption effects (for example, Raman spectroscopy spectra are usually accompanied by a strong background due to fluorescence and elastic scattering^6,35^), and much more. Last, measurements are also affected by noise that sometimes play a relevant role in the quality of the measurement (typical examples are some Raman spectroscopy applications where the Raman light is of the order of the noise and differential absorption lidar where the noise define the maximum range and the sensitivity of the technique^36^). Thus, the previous equation may be written as *y* = *g(I*_*d*_*, I*_*b*_*, I*_*n*_*)*, where *I*_*b*_ is what will be named in the article as “background noise”, which represents all the effects due to other phenomena*, I*_*n*_ is the noise and the function *g* is of course different from the physical known function *f*.

In general, some countermeasures can be taken to address such problems. For example, noise is usually addressed by reducing the noise due to the instruments and by performing statistics, when possible, by averaging several spectrum measurements. The background is also usually faced instrumentally, for example by filtering out the wavelengths that contain only the background radiation.

However, even reducing such effect, it is usually required to find the function g that allows to evaluate our desired information y and the only way to find this function is very often based on data-driven approaches that is typically known as calibration^37,38^. A calibration can be done in different ways. The most simple and widespread method is based on performing controlled measurements of I (where I is the measured spectrum due to *I*_*d*_*, I*_*b*_*,* and* I*_*n*_*)* where y is known and by a fitting approach one can find the function that allows to evaluate y from I^39^. When the problem is quite complex, it may be hard to find a simple equation (polynomial, power law, etc.) that fits the data, and therefore more complex approaches are used. Today, one of the most utilised methodologies is supervised machine and/or deep learning. The rationale is very similar to function fitting, since the machine learning algorithm is a very general function (considering as machine learning tool a sufficiently deep method, such as neural networks or decision trees), that it is “trained” with labelled data, i.e. measurements where both the input (in our case I) and the output (y) are known. These artificial intelligence methods are very powerful and usually they far exceed by far the classical calibration method^40–45^.

While a ‘supervised’ method is the best approach to perform a calibration^46,47^, it may sometimes be impossible to be applied. For example, sometimes it is not viable to reproduce controlled experiments where *y* is a priori known and, therefore, the training phase (or the fitting) is impractical to perform. In such cases, only theoretical assumptions based on the physics of the phenomenon can help to find an acceptable calibration function.

In this paper, a new unsupervised deep learning calibration methodology based on Physics-Informed Neural Network (PINN) is introduced. Physics-Informed Neural Network^48,49^, or PINN, is an emerging machine learning technology that is gaining attention in all fields of science (and recently also industries). The main difference from classic supervised machine learning is the way they are trained, since they do not require the knowledge of the output to be trained but use physical expectation. Contrary to standard machine learning, that uses expected vs actual output based loss functions, the physics-informed machine learning aims at minimising a loss function that looks at the physics. Typically, PINNs have been used to simulate flows (Navier–Stokes equations)^50,51^, and solve specific PDEs^48^, but are now finding applications also in various other sectors such as medicine^52^ and energy^53^.

In this work, we present a new way to use PINN to develop a methodology that can calibrate or evaluate specific information from spectra without the need to use a supervised method. This new approach is of course interesting for all those cases where it is impossible to perform a typical calibration or train a supervised machine learning algorithm, since a “training set” cannot be produced.

The paper is divided into sections as follows. Section “Unsupervised physics-informed calibration” aims at showing the methodology used, by introducing the general calibration problem and proposing a new methodology to evaluate the information that can be extracted from the spectra. Section “Results” shows the results of the new method by analysing the performances in numerical (or synthetic) cases and one experimental case reproduced in our laboratory. Section “Discussion and conclusions” is dedicated to discussion, conclusion, and future developments, while in Section “Materials and methods” it is possible to find all the details regarding the materials and methods to replicate this work.

## Unsupervised physics-informed calibration

Consider a light-matter interaction that produces a spectrum *I*_*d*_*(λ)* as a function of the concentration *c* of a specific element such that:

1 $${I}_{d}\left(\lambda \right)=g\left(c,\lambda \right),$$

where *g(c, λ)* is a general function that describes the intensity of the spectrum generated at each wavelength λ once the concentration *c* is defined. For the sake of simplicity, suppose that there is linear functionality between *I*_*d*_*(λ)* and *c*, so the previous function can be written as:

2 $${I}_{d}\left(\lambda \right)={I}_{d,0}\left(\lambda \right)c,$$

where *I*_*d,0*_*(λ)* is the spectrum when *c* is equal to one, which will be named “reference spectrum” from now on. Suppose also that during the measurement other effects occur such that the recorded spectrum *I(λ)* is the sum of *I*_*d*_*(λ)*, *I*_*b*_*(λ)*, and *I*_*n*_*(λ)*, where *I*_*b*_*(λ)* is a variable spectrum due to other effects (the “background spectrum”) and *I*_*n*_*(λ)* is a random spectrum due to the noise (electrical, optical, etc.). The only way to measure *c* (without using supervised learning) is to estimate *I*_*b*_*(λ)*, so that it can be removed from *I(λ)* and Eq. (1) can be applied (*I*_*d,0*_*(λ)* is considered known). In this work, we present an innovative method to estimate *I*_*b*_*(λ)*, and thus calculate *I*_*d*_*(λ)* e *c*, by using a physics-informed neural network.

**Figure 1:**
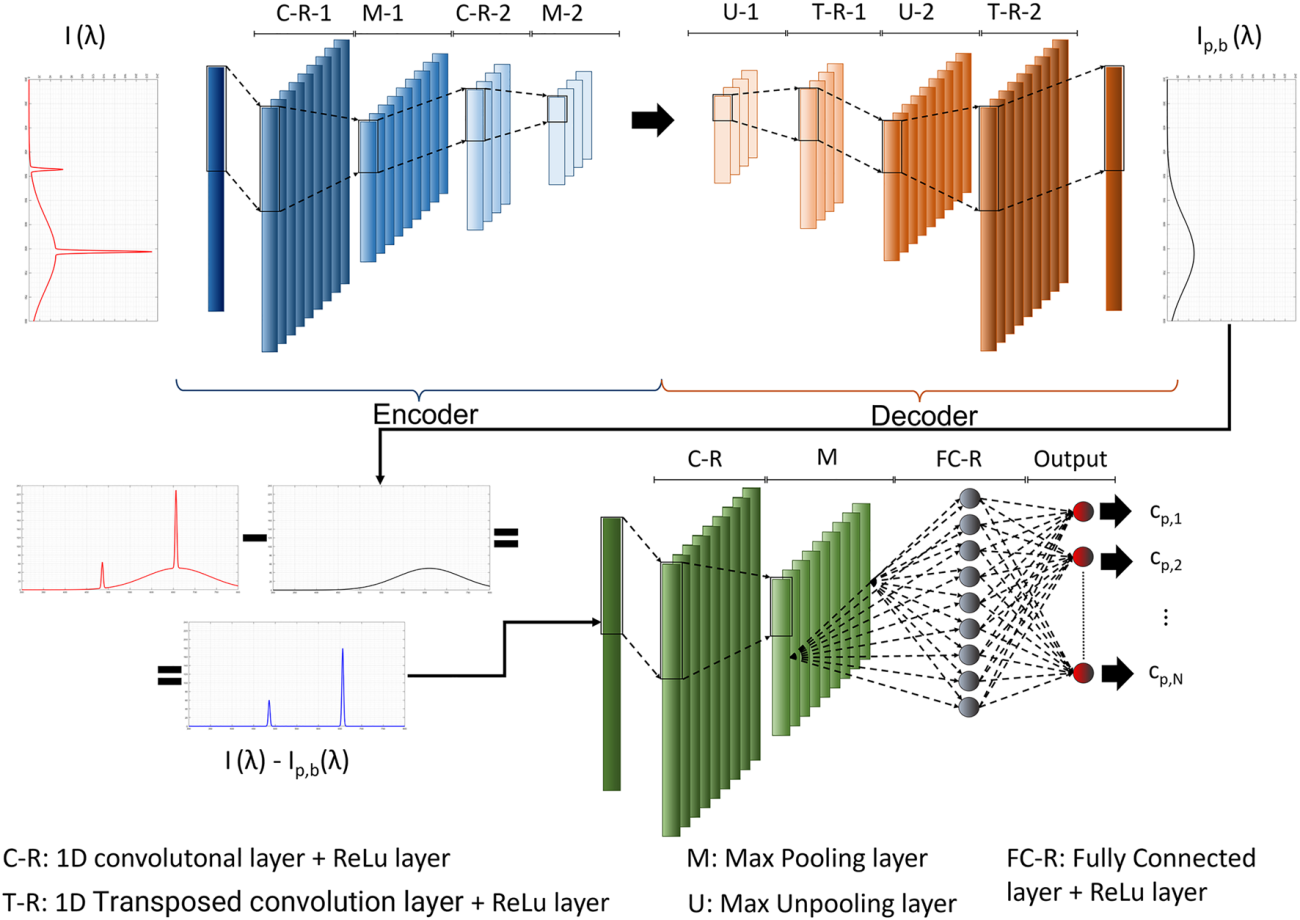
Physic-Informed Neural Network (PINN) architecture used in this work. The top shows the autoencoder that aims to predict the background spectrum, while the bottom shows the net that aims to predict the concentration.

The PINN architecture, described in detail in Section “Materials and methods”, is shown in Fig. 1. A typical autoencoder structure^54^ is applied to the input, the measured spectrum *I(λ)*, with the aim of evaluating the background noise *I*_*p,b*_*(λ)*. Thus, the predicted background is removed from the measurement and a new convolutional layer (with the associated MaxPooling and ReLu layers) is applied. Then, a fully connected layer is used to predict the concentration *c*_*p*_.

The neural network is based on minimising a loss function composed of two terms. The first is the reconstruction error. In fact, we expect that the sum of the predicted background spectrum and the predicted I_p,d_, calculated as the reference spectrum (I_d,0_(λ)) multiplied by the predicted concentration (c_p_) is equal to the measured one I(λ) (excluding the error associated with the noise, assumed to be averagely zero. Please note that all the systematic errors will be automatically computed in the background noise):

3 $${L}_{rec}=\sum {\left(I\left(\lambda \right)-{I}_{p,d}\left(\lambda \right)-{I}_{p,b}\left(\lambda \right)\right)}^{2}=\sum {\left(I\left(\lambda \right)-{c}_{p} {I}_{d,0}\left(\lambda \right)-{I}_{p,b}\left(\lambda \right)\right)}^{2}.$$

However, this solution is not sufficient to find a unique solution (the PINN may always choose to predict ${I}_{p,b}\left(\lambda \right)=I(\lambda )$ and ${c}_{p}=0$). Therefore, a regularisation term has been added:

4 $${L}_{reg}=\sum {\left(\frac{d{I}_{p,b}}{d\lambda }\right)}^{2}.$$

This term asks the PINN to avoid background spectra when possible, *i.e.* when the shape of the measured spectrum fits *c*_*p*_*I*_*0*_*(λ)*. Thus, the loss term is written as follows:

5 $${L}_{tot}={L}_{rec}+\alpha {L}_{reg}=\sum {\left(I\left(\lambda \right)-{c}_{p} {I}_{d,0}\left(\lambda \right)-{I}_{p,b}\left(\lambda \right)\right)}^{2}+\alpha \sum {\left(\frac{d{I}_{p,b}}{d\lambda }\right)}^{2},$$

where *α* weights the importance of the regularisation term. As usual, the regularisation term plays a relevant role in the quality of the algorithm. In Section “Deep learning architecture”, we provide a methodology to estimate a good value of *α* for this neural network. However, it is worth to highlight that is a good practice to train the algorithm with different regularisation values and by inspecting the results critically.

The previous methodology, which has been shown for one interesting emitting agent (or class), can be easily extended to a multi-agent (multiclass) reconstruction. In that case, the architecture of the net is the same, except the output layer that predicts two or more concentrations. The loss function is modified as follows:

6 $${L}_{tot,multi\,class}={L}_{rec}+\alpha {L}_{reg}=\sum {\left(I\left(\lambda \right)-\sum_{j=1}^{N}{c}_{p,j} {I}_{0,j}\left(\lambda \right)-{I}_{p,b}\left(\lambda \right)\right)}^{2}+\alpha \sum {\left(\frac{d{I}_{p,b}}{d\lambda }\right)}^{2},$$

where *c*_*p,j*_ is the concentration and *I*_*0,j*_ is the reference spectrum of the j-th agent respectively and N is the number of agents.

Another consideration regards the linearity assumption. In fact, at the beginning of this section, we assumed that the process is working in a linear regime, a hypothesis that is not always satisfied. In such a case, the linear function should be substituted with the non-linear one and a more general loss function becomes:

7 $${L}_{tot,multi\,class}={L}_{rec}+\alpha {L}_{reg}=\sum {\left(I\left(\lambda \right)-\sum_{j=1}^{N}{g}_{j}\left({c}_{p,j},\lambda \right)-{I}_{p,b}\left(\lambda \right)\right)}^{2}+\alpha \sum {\left(\frac{d{I}_{p,b}}{d\lambda }\right)}^{2},$$

where *g*_*j*_*(c*_*p,j*_*,λ)* is the function to estimate the expected spectrum *I*_*d,j*_ from the concentration *c*_*p,j*_ see Eq. (1).

## Results

The proposed methodology has been tested with both synthetic and experimental datasets. At first, we show the synthetic cases that allowed us to better investigate the advantages and the limits of the new methodology. Then, an experimental case with measurements performed in our laboratory is analysed.

### Synthetic datasets

Synthetic tests have the advantage of controlling all the variables and ensure that the hypotheses at the basis of the algorithm are fulfilled. Six datasets have been generated to test the algorithm at different difficulties:

1. N01: Peaked reference spectrum with a steady constant background;
2. N02: Peaked reference spectrum with a unsteady constant background;
3. N03: Peaked reference spectrum with a steady shaped background;
4. N04: Peaked reference spectrum with a unsteady shaped background;
5. N05: Not peaked reference spectrum with a unsteady shaped background;
6. N06: Two classes peaked reference spectra with an unsteady shaped background.

**Figure 2:**
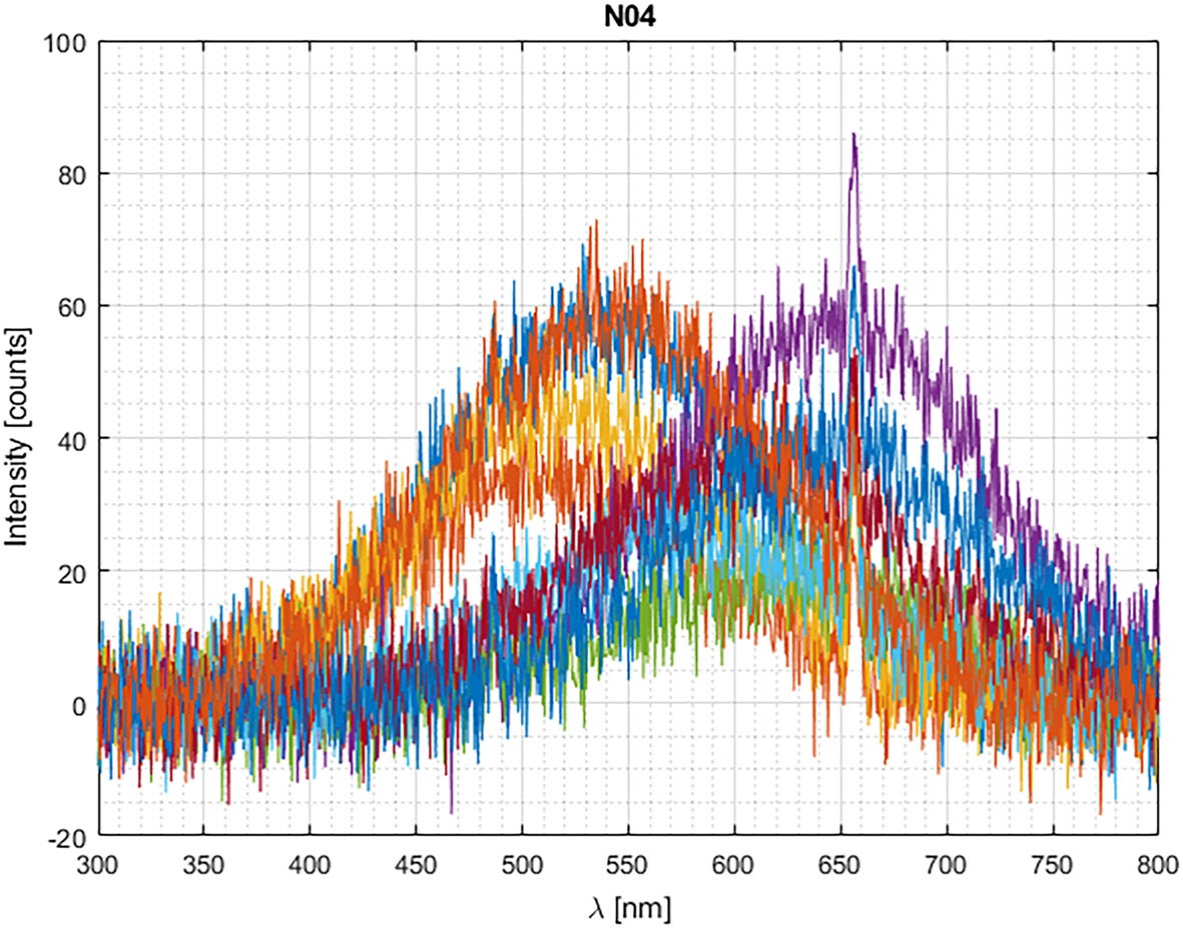
Example of spectra in the N04 dataset, showing spectra calculated with agent concentrations ranging from 0.1 and 0.15. The image aims to show in what extreme conditions the algorithm has been tested.

In the case of N01 and N02, the use of a deep learning is not justifiable since a simple offset allows to ensure the highest performances. However, for the sake of completeness and to show that the method works also for these simple cases, these datasets have been analysed and the performances have been compared with a simple offset removal approach. Also, in the case of N03 the deep learning method proposed may be not needed since after a statistical analysis one may be able to automatically remove the background and analyse the polished spectrum. Things worsen in the case of N04, where the background strongly varies from measurement to measurement, involving that the statistics becomes complex, and it is not easy to maximise its efficiency. N05 analyses the case of a reference spectrum that does not reach a peak, making the analysis more challenging since the shapes of the background and the reference are quite similar. N06 validates the multiclass approach under the same conditions as N04. A detailed description of the functions used to generate the synthetic spectra is provided in Section “Materials and methods”. Just to give an idea of the complexity of the task, the “measured spectra” with concentrations ranging between 0.1 and 0.15 are provided in Fig. 2. In this figure, the part of the spectrum related to our agent is represented by the peaks at 656 nm and 486 nm. The graph clearly shows that the variability of the background is very large compared to the intensity of the two peaks, involving the automatic separation of the spectrum due to the agent from the background spectrum being not an easy task.

**Figure 3:**
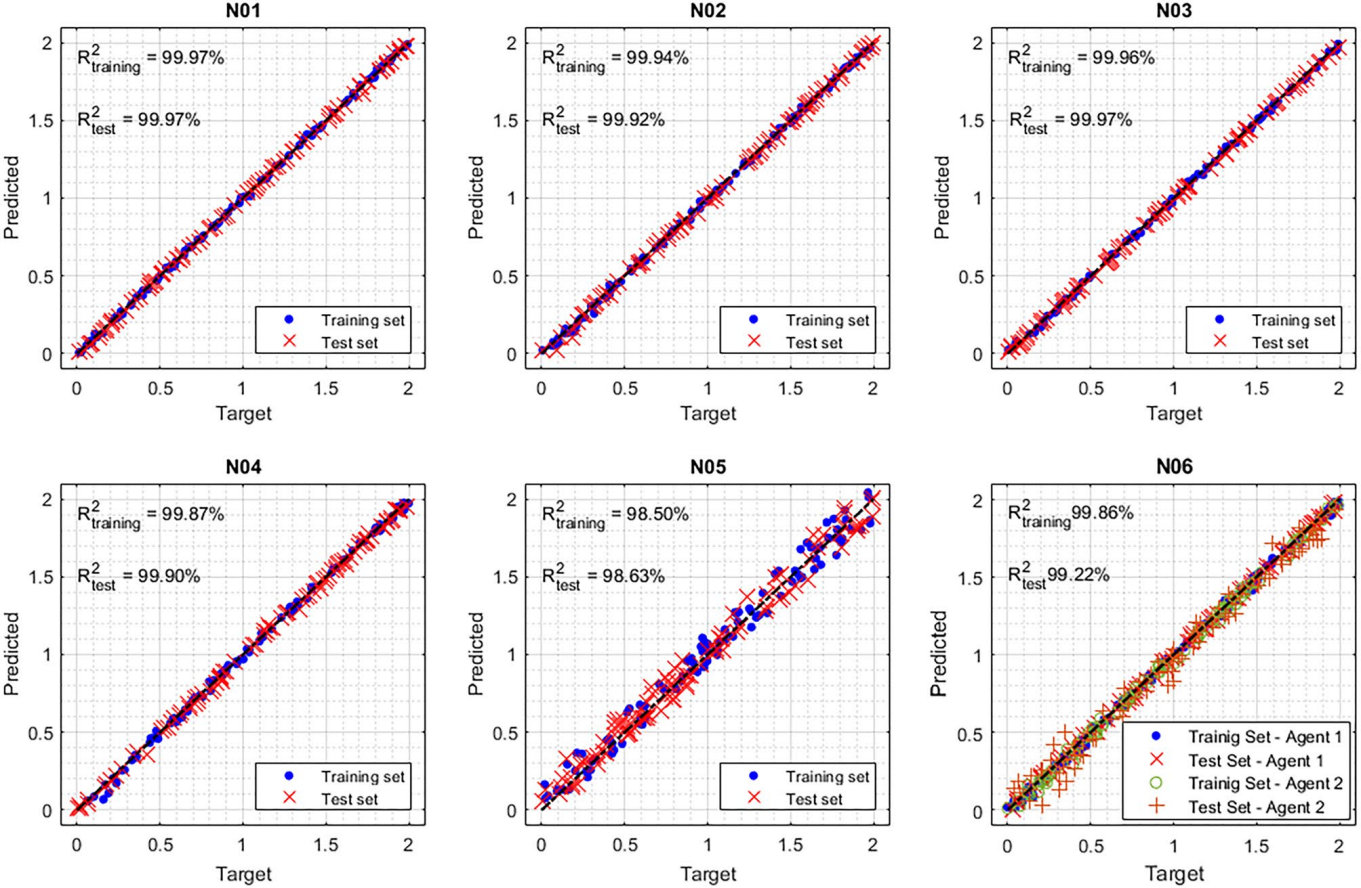
Results obtained in the six synthetic cases. Each figure shows the target concentration vs the predicted concentration for the six synthetic cases for both training and test datasets.

For each case, the PINN has been trained on a set of data (“training set”) and has been evaluated on new data (“test set”). Despite the fact that the PINN cannot overfit the data (it does not use supervised training), we decided to test them also with new data (test set) to demonstrate that, once the net is trained, it can also be easily used for real-time applications. The results of these six cases (from N01 to N06) are shown in Fig. 3. Both the training and test target vs predicted data are shown and the R-squared is displayed. Each graph shows the target concentration (i.e. the one used to generate the data) vs the predicted concentration by the PINN. For very simple situations (N01, N02), the prediction is practically perfect. This good result is also observed for more challenging cases (N03, N04), while a slight decrease in performances is observed in the most challenging cases (N05, N06). However, it has to be highlighted that also in these difficult cases, the relative prediction errors were below 1%. These results clearly demonstrate that the application of PINN allows us to obtain very accurate results when the physical phenomena is known.

### Experimental datasets

Experimental tests have been performed on Laser-Induced Fluorescence spectra. The authors set up an experiment in which samples of riboflavin (B2 vitamin) were irradiated with a 280 nm power LED. The emitted fluorescence at larger wavelengths (typically from 450 to 700 nm) is recorded at 90° through a spectrometer. To simulate variable background radiation, an intensity-variable tungsten lamp has been used. By varying the voltage of the lamp, the intensity (and slightly the shape) of the background radiation has been varied from measurement to measurement. A detailed description of the experimental apparatus, procedure, and typical measured spectra is reported in the “Materials and methods” section.

**Figure 4:**
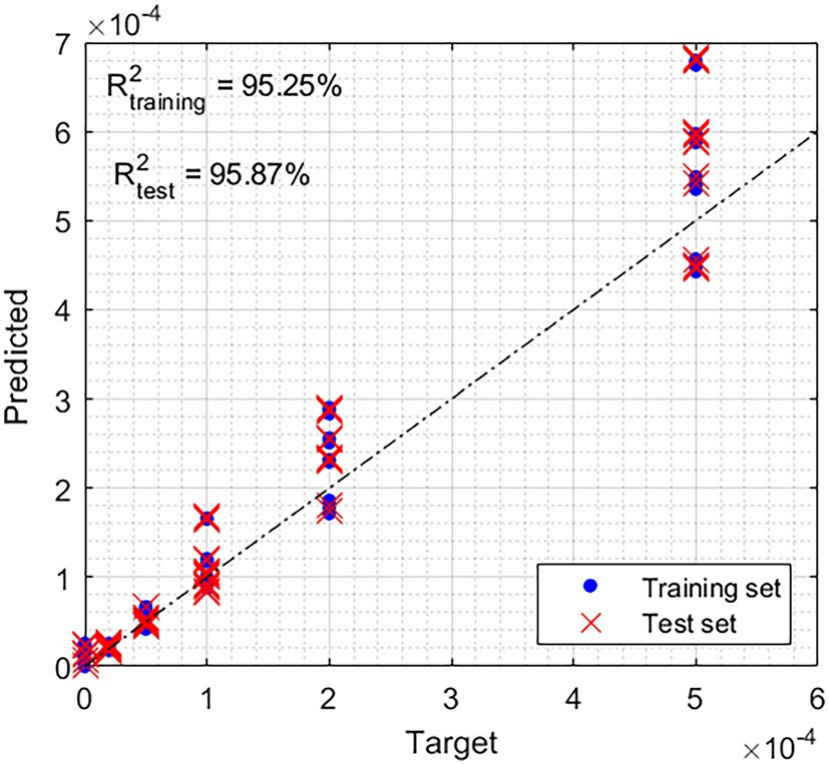
Results obtained on experimental Laser-Induced Fluorescence spectra. The figure shows the target vs predicted concentrations for both training and test datasets.

The results are shown in Fig. 4. Also, in this case, the target concentration (estimated by measuring the mass of riboflavin used to make the sample) is compared with the predicted one. It can be seen that, in this case, the reconstruction quality is worse compared to the synthetic cases. In this case, the concentration prediction error is on average about 10%. This larger error is mainly due to three reasons:

1. The fluorescence and background spectra are continuous and similar. This involves a larger confusion. It is clear that in the case where the background and reference spectra are equal, inversion is not possible (this is also true for the supervised calibration).
2. The target concentrations are affected by uncertainties (even if they should be smaller than 5%).
3. For this experiment, the linear reconstruction loss function was used Eq. (3). However, our spectrometer shows a clear non-linear behaviour (the counts do not increase linearly with the input intensity). This involves that the measured spectrum is distorted with respect the reference one, and concentrations are systematically overestimated or underestimated by a value proportional to the non-linearity.

## Discussion and conclusions

A new physics-informed deep learning method has been developed to calibrate spectra without the need for dedicated calibration experiments. The method consists of finding the concentrations of the desired species by reconstructing the measured spectra. Contrary to classic calibrations, that require the set of measurements where the output is known, this approach does not require a dedicated set but the knowledge of the physics of the phenomenon. This unsupervised method can play a relevant role in all situations where standard calibration methodologies and supervised machine learning algorithms are not deployable (while in these other cases, it is better to use the standard methodologies, as supervised methods are more accurate than unsupervised).

A second relevant feature of this method is that it allows the decoupling of the effects composing the spectra. In fact, the first part of the neural network aims at finding the background, which in some cases can contain useful information about the physical phenomenon. For example, in laser-induced fluorescence when a fourth harmonic Nd:YAG wavelength is used to induce the fluorescence, the second harmonic scattering is usually present in a spectral region where also fluorescence is present (532 nm). By using our method, we may clean the fluorescence spectrum from the scattering one, without the necessity to filter the signal through optical filters.

Generally speaking, the methodology that we presented in this paper can have several applications in optics, from calibration of diagnostics (that is the case study used to introduce the methodology) to cleaning of spectra and the solution of inverse problems.

As with all the supervised machine learning techniques, once the net is trained, it can be easily run in real-time. By analysing the average time required by our neural network on new data (statistics performed on 6000 samples), it has been found that the average prediction time is 10 ms, with a minimum value of 7 ms and a maximum one of 15 ms. Even if this prediction time is compatible with most real-time applications, it is worth mentioning that the tests have been performed on a simple laptop with a code that it has not been optimised for real-time prediction. It is expected that the prediction speed can be easily reduced by, at least, an order of magnitude.

It is also worth mentioning that this methodology can be integrated with a sort of adaptive training. In fact, while standard calibration requires the knowledge of the output, and therefore it is trained on ad hoc experiments, the physics informed can be trained on all experiments. Therefore, new experiments mean new data for PINN training, allowing the extension of the ‘training set’, the “evolution” of the neural network, and therefore a network with a wide range of applicability.

A fairly general regularisation loss function for similar work has been developed. However, readers are strongly encouraged to perform parametric analysis to evaluate the best regularisation term. It is also worth mentioning that this methodology may be implemented with other physics-guided ad hoc loss functions, developed for specific cases to ensure higher performance. In fact, as this methodology based on the knowledge of the physics of the phenomenon, it is clear that it is impossible for the authors to develop loss functions that work in all areas of optics. Thus, the methodology shown in this paper should be considered as a recipe to develop ad hoc neural network architectures and loss functions to optimise specific problems.

Of course, such a powerful methodology has its limits. First, it is crucial to know the physics of the phenomenon (the ‘reference spectra’ or, in general, the *g(c,λ)*). Moreover, the responses of the instrumentation must be known. Non-linearities, cut-offs, etc. must be taken into account and written in the loss functions; otherwise, systematic errors would arise. In our experimental case, for example, the non-linearity of our spectrometer leads to large errors (see “Experimental results” section).

It is worth highlighting that the methodology shown in this work has been used without any type of pre-processing tool, method, or analysis. However, it is clear that the use of some preprocessing algorithms to better select the input data before the training may help in ensuring a better training set, resulting in more performant algorithms. Typical procedures may be smoothing techniques or the averaging of more measurements, but also the use of some advanced processing tools, such as those based on fuzzy logic, that allow data association without supervision^55–57^.

In conclusion, a new methodology combining physics knowledge and artificial intelligence has been developed to automatically evaluate some quantities from spectra without the need to supervise the machine learning tool. It is obvious that this approach may lead to huge improvements in all the scientific and industrial cases where ad hoc calibration measurements cannot be performed or the actual values are not accurately defined.

Moreover, even if this methodology has been shown for optical spectroscopic measurements, it has to be highlighted that the approach may be transferred not only other spectroscopic measurements, but it may be deeply generalised to be potentially applied, by changing the neural network architecture and the loss function, to any type of measurements that require a calibration procedure. The future developments of this work go exactly in this direction, since we will try to generalise the methodology for other physics and engineering cases.

## Materials and methods

In this section, the details concerning the neural network, the synthetic cases generated for the test, and the experimental setup to collect the LIF spectra are described.

### Deep learning architecture

The architecture of our physics-informed deep learning algorithm is shown in Fig. 1. The input is a spectrum, and, by an autoencoder-like structure, the background is predicted. Autoencoders are usually divided into Encoder and Decoder. The encoder is composed of four layers: two convolutional layers followed by two MaxPool layers^58^. After each convolutional layer, a ReLU (Rectified Linear Unit) layer is used. The output of the code is then deconvoluted through the decoder, which has two deconvolutional and MaxUnPool layers. Deconvolutional layers are followed by a ReLU layer each. The output of the decoder is the predicted background spectrum.

Then, the predicted background spectrum is removed from the measured spectrum, and a convolutional layer, followed by a ReLU and MaxPool are applied. Therefore, a fully connected layer is applied and the output layer returns the net output. The transfer function of the fully connected layer is a ReLU, while the output layer transfer function is a linear one. Several attempts have been made to “tune” the hyperparameters of the neural network architecture (convolutional size, fully connect size, etc.) and the chosen hyperparameter is resumed showed in Table 1.

**Table 1:** Neural Network Hyperparameters.

	Neural network architecture tests on datasets from N01 to N06				
	FC neurons	Kernel	Number of filters	Pool size	Stride
C-R-1	–	[20 1]	20	–	–
M1	–	–	–	10	10
C-R-2	–	[20 1]	7	–	–
M2	–	–	–	14	14
T-R-1	–	[20 1]	7	–	–
T-R-2	–	[20 1]	20	–	–
C-R	–	[20 1]	5	–	–
M	–	–	–	50	100
FC-R	10	–	–	–	–
FC-output	1, 2 (As a function of output concentrations)	–	–	–	–

Please refer to Fig. 1 for layer labels.

The loss function is described in the main text, and no additional information must be provided.

For what concerns the test performed on the datasets from N01 to N05, the number of batches is 100 while the number of epochs is 5 10^3^. For the N06 dataset, the number of epochs equal is 5 10^4^. Instead, to test the algorithm on the real case, the batch number has been set equal to 60 with a number of epochs equal to 25 10^4^. The training algorithm is ADAM^59^.

For our cases, no significant differences have been observed for most hyperparameters. Of course, an important role is played by the code size (maximum compression of the data). It has been found that, for our synthetic cases, a 5 × 7 size is usually enough (increasing the size no improvements have been observed), for both one-class and double-class tests, while the experimental cases we used a 2 × 20 code.

Another hyperparameter is the regularisation term (α in Eq. (5)). As usual, there is no magic number, and it is strongly suggested to try several tries before accepting a result. In our case, we found that a good solution is:

8 $$\alpha =\frac{1}{\Delta \lambda }\frac{\sum {I}_{0}}{\sum \left|\frac{\partial {I}_{0}}{\partial \lambda }\right|}$$

This number has been derived just by imposing two conditions, the first is that the relative reconstruction error should be small, and the second is that the background spectrum should contain smoother features in respect to reference spectra (these conditions are satisfied in several conditions, since the background is the sum of several effects that tend to smooth the resulting total spectrum). However, we would like to highlight again that even if this number provided to be a very good choice for our cases, a parametric analysis should always be performed to select the best regularisation term.

### Synthetic approach: datasets and tests

Six synthetic tests have been performed:

1. N01: Peaked reference spectrum with a steady constant background;
2. N02: Peaked reference spectrum with a unsteady constant background;
3. N03: Peaked reference spectrum with a steady shaped background;
4. N04: Peaked reference spectrum with a unsteady shaped background;
5. N05: Not peaked reference spectrum with a unsteady shaped background;
6. N06: Two classes peaked reference spectra with an unsteady shaped background.

The “measured” spectra have been calculated by generating a reference spectrum, random concentrations ranging from 0 to 2, background and noise spectra, and summing up all of them.

In the case of the reference spectrum for one species (N01, N02, N03, N04, N05), I_0_ has been calculated as follows (parameter values are shown in Table 2):

**Table 2:** Values used to generate the reference spectra.

			N01			N02			N03			N04			N05		
I_1_			180			180			180			180			180		
I_2_			60			60			60			60			60		
λ_1_			656.3			656.3			656.3			656.3			656.3		
λ_2_			486.1			486.1			486.1			486.1			486.1		
σ_1_			2			2			2			2			45		
σ_2_			2			2			2			2			40		
N06	I_1,1_	I_1,2_		I_2,1_	I_2,2_		λ_1,1_	λ_1,2_		λ_2,1_	λ_2,2_		σ_1,1_	σ_1,2_		σ_2,1_	σ_2,2_
	180	60		240	100		656.3	486.1		587.5	480.0		2	2		2	2

9 $${I}_{0} = {I}_{1}{e}^{-\left(\frac{{\left(\lambda -{\lambda }_{1}\right)}^{2}}{{\left(2{\sigma }_{1}\right)}^{2}}\right)}+{I}_{2}{e}^{-\left(\frac{{\left(\lambda -{\lambda }_{2}\right)}^{2}}{{\left(2{\sigma }_{2}\right)}^{2}}\right)}$$

While in the case of two species:

10 $$\begin{gathered} I_{0,species\,1} =I_{1,1} e^{{ - \left( {\frac{{\left( {\lambda - \lambda_{1,1} } \right)^{2} }}{{\left( {2\sigma_{1,1} } \right)^{2} }}} \right)}} + I_{1,2} e^{{ - \left( {\frac{{\left( {\lambda - \lambda_{1,2} } \right)^{2} }}{{\left( {2\sigma_{1,2} } \right)^{2} }}} \right)}} \hfill \\ I_{0,species\,2} = I_{2,1} e^{{ - \left( {\frac{{\left( {\lambda - \lambda_{2,1} } \right)^{2} }}{{\left( {2\sigma_{2,1} } \right)^{2} }}} \right)}} + I_{2,2} e^{{ - \left( {\frac{{\left( {\lambda - \lambda_{2,2} } \right)^{2} }}{{\left( {2\sigma_{2,2} } \right)^{2} }}} \right)}} \hfill \\ \end{gathered}$$

The background spectra are generated as follows:

11 $$\begin{gathered} {\text{N}}01: \,I_{back} \,\left( \lambda \right)\, = \,10 \hfill \\ {\text{N}}02: I_{back} \left( \lambda \right) = 10\varepsilon\, where\, \varepsilon\, is\, a\, random\, number\, from\, 1\, to\, 6 \hfill \\ {\text{N}}03:{ }I_{back} \left( \lambda \right) = 50e^{{ - \left( {\lambda - 500} \right)^{2} /\left( {2*75^{2} } \right)}} \hfill \\ {\text{N}}04,\,{\text{N}}05,{\text{ N}}06: I_{back} \left( \lambda \right) = 50\varepsilon_{1} e^{{ - \left( {\lambda - 500 - 200\varepsilon_{2} } \right)^{2} /\left( {2*75^{2} } \right)}} \hfill \\ where\, \varepsilon_{1} \,is\, a\, random\, number\, from\, 1 \,to\, 6\, and\, \varepsilon_{2} \, is\, a\, random\, number\, from\, 0 \,to\, 1 \hfill \\ \end{gathered}$$

Random noise has been added by generating a random number normally distributed with zero mean and standard deviation equal to five.

### Real-case potential used tests: datasets and tests

Experimental tests have been performed by irradiating samples of riboflavin with ultraviolet (UV) light. The fluorescence spectra have been recorded by a spectrometer. The light source is a 280 nm centred wavelength diode. A laser line filter with a central wavelength equal to 280 nm and an aperture of 20 nm (± 10 nm) has been used to filter out the long radiation tale of the diode. The spectrometer is a Flame from the Ocean Insight company. It has 2048 pixels, with a working wavelength range from 180 to 874 nm. The average resolution is 0.33 nm. The exposure time for all measurements was 2 s. In order to simulate undesired background radiation, an intensity-variable tungsten lamp has been used. By varying the voltage, the intensity and the shape of the spectrum of the background vary. During the various measurements, the voltage (and therefore the intensity) has been varied to ensure a high variability of the background. However, since the fluorescence is observed from about 450 nm, the spectra have been cut-off for λ <  = 400 nm.

Several riboflavin samples have been prepared at different concentrations, by diluting riboflavin in distilled water:

- Sample 1 = 5 10^–4^ g/l
- Sample 2 = 2 10^–4^ g/l
- Sample 3 = 1 10^–4^ g/l
- Sample 4 = 5 10^–5^ g/l
- Sample 5 = 2 10^–5^ g/l
- Sample 6 = 0 g/l

In total, 240 spectra have been recorded with variable background. Half of them have been used for the training, while the other 120 have been tested (see Fig. 4). The reference spectrum has been evaluated by measuring the LIF spectra of riboflavin in the absence of background radiation (calculate as the average of 10 spectra to reduce the presence of the noise in the reference one). The riboflavin concentration for the reference spectrum was 10^–4^ g/l.

## Data Availability

The experimental measurements and the codes to generate the synthetic spectra and train the PINN will be provided by the author upon request (contact: r.rossi@ing.uniroma2.it).
